# Das FH-defiziente Nierenzellkarzinom erweitert das Spektrum der papillären Tumoren in der Niere

**DOI:** 10.1007/s00292-021-00977-y

**Published:** 2021-08-27

**Authors:** N. Rupp, H. Moch

**Affiliations:** 1 Institut für Pathologie und Molekularpathologie, Universitätsspital Zürich, Schmelzbergstr. 12, 8091 Zürich, Schweiz; 2 Universität Zürich, Zürich, Schweiz

**Keywords:** Citratzyklus, Differenzialdiagnose, Fumarat-Hydratase, Hereditäre Leiomyomatose und Nierenzellkarzinom, Mutation mit Funktionsverlust, Citric acid cycle, Differential diagnosis, Fumarate hydratase, Hereditary leiomyomatosis and renal cell cancer, Loss of function mutation

## Abstract

Das Fumarat-Hydratase(FH)-defiziente Nierenzellkarzinom (NZK) ist eine distinkte Entität, welche eine biallelische Inaktivierung des *FH*-Gens zeigt, die konsekutiv mit einem Expressions- bzw. Funktionsverlust des FH-Proteins einhergeht. Diese Alteration führt zu einer Akkumulation des Onkometaboliten Fumarat im Citratzyklus und vielfältigen Störungen des Zellhaushaltes und der DNA-Prozessierung. Das FH-defiziente NZK zeigt häufig ein morphologisch überlappendes Spektrum mit papillären NZK (Typ 2), wobei typischerweise ein Wechsel verschiedener Wachstumsmuster inkl. tubulozystischer, kribriformer und/oder solider Differenzierung zu beobachten ist. Eine typische, jedoch nicht spezifische morphologische Eigenschaft sind die prominenten eosinophilen, Viruseinschlußkörperchen-artigen Nukleolen mit perinukleolärem Halo. Der immunhistochemische Verlust der FH-Expression untermauert die Diagnose, kann in seltenen Fällen jedoch erhalten sein. Zumeist zeigen FH-defiziente NZK ein sehr aggressives biologisches Verhalten mit oftmalig primärer Metastasierung bei Diagnosestellung. Die initiale Beschreibung erfolgte als NZK in Assoziation mit dem Hereditären-Leiomyomatose-und-Nierenzellkarzinom(HLRCC)-Syndrom, welches zusätzlich kutane und uterine Leiomyome umfasst. Aktuelle Daten zeigen jedoch auch einen steigenden Anteil an sporadischen Fällen, sodass eine Unterscheidung (hereditär vs. sporadisch) angemessen erscheint. Bisher sind wenige, aber vielversprechende Daten bezüglich wirksamer systemischer therapeutischer Optionen beschrieben. Zusammenfassend ist eine korrekte Diagnose aufgrund des typischerweise biologisch aggressiven Verhaltens, gegebenenfalls vom Standard abweichender therapeutischer Optionen und möglichem Indikator einer hereditären Erkrankung von großer Bedeutung.

## Abstract (en)

Fumarate hydratase (FH)-deficient renal cell carcinoma (RCC) is a distinct entity, which shows a biallelic inactivation of the *FH* gene that consequently leads to FH protein expression and function loss, respectively. This alteration leads to an accumulation of the oncometabolite fumarate in the citrate cycle and various disorders of the cell balance and DNA processing. FH-deficient RCC often shows a morphologically overlapping spectrum with papillary renal cell carcinoma (type 2), whereby a typical mixture of growth patterns including tubulo-cystic, cribriform, and/or solid differentiation can be observed. A characteristic but non-specific morphological feature is prominent eosinophilic, virus-inclusion body-like nucleoli with perinucleolar halos. Tumoral immunohistochemical loss of FH expression supports the diagnosis but may be preserved in rare cases. Most FH-deficient RCCs show very aggressive biological behavior and are often metastasized at the time of diagnosis. The initial description encompassed RCC in association with the hereditary leiomyomatosis and renal cell carcinoma (HLRCC) syndrome, which also includes cutaneous and uterine leiomyomas. However, current data also show an increasing proportion of sporadic cases, so that a distinction (hereditary vs. sporadic) seems appropriate. So far, few but promising data on effective systemic therapeutic options have been reported. In summary, precise diagnosis is of great importance due to the frequent aggressive biological behavior, potential need to deviate from the therapeutic standard, and the possible indicator of a hereditary disease.

Das Fumarat-Hydratase(FH)-defiziente Karzinom der Niere wurde aufgrund der häufig großen Ähnlichkeiten zu papillären Nierenzellkarzinomen (NZK) früher typischerweise im Spektrum papillärer NZK Typ 2 klassifiziert. In der WHO-Klassifikation 2016 wurden diese Tumoren erstmals als separate Entität unter dem Namen der Hereditären-Leiomyomatose-und-Nierenzellkarzinom(HLRCC, OMIM: #150800)-Syndrom-assoziierten Nierenzellkarzinome (HLRCC-RCC) klassifiziert [1]. Die Diagnose beruht dabei auf dem typischen Expressionsverlust des Citratzyklus-assoziierten Proteins Fumarat-Hydratase in den Tumorzellen, welcher mittels Immunhistochemie dargestellt werden kann [2].

## Geschichte

Im Jahr 2001 wurden erstmals 2 finnische Familien beschrieben, in denen die Assoziation von uterinen und kutanen Leiomyomen sowie Nierenzellkarzinomen auftraten [3]. Im folgenden Jahr konnte bereits die Verbindung zum Gen *Fumarat-Hydratase* (*FH*) bzw. dem davon codierten Protein als Tumorsuppressor hergestellt werden [4]. Der Erbgang erfolgt in der heterozygoten Konstellation autosomal-dominant, wohingegen bestimmte (angeborene) homozygote *FH*-Mutationen (autosomal-rezessiv) mit starken psychomotorischen Einschränkungen, Hirnfehlbildungen und Tod in der ersten Lebensdekade beschrieben sind (Fumarazidurie: OMIM #606812) [5]. In der autosomal-dominanten (heterozygoten) Konstellation eines Tumorsyndroms wurde das Konzept des somatischen „second hits“ mit einem „loss of heterozygosity“ des Wildtypallels bereits zum Zeitpunkt der Erstbeschreibung entwickelt [6]. Aufgrund der initialen Entdeckung im Rahmen einer hereditären Assoziation wurden diese Nierenzellkarzinome unter dem Namen des Hereditäre-Leiomyomatose-und-Nierenzellkarzinom-Syndrom-assoziierten Nierenzellkarzinoms (HLRCC-RCC) erstmals in die international gültige WHO-Klassifikation für Tumoren des Harntraktes und der männlichen Geschlechtsorgane aufgenommen [1]. Aufgrund neuerer Daten, die auch einen nicht unerheblichen Anteil von sporadischen Fällen (bis 16 %) derartiger Nierenzellkarzinome zeigen [7], deutet sich in der Literatur ein Wechsel hin zum allgemeingültigen Namen des FH-defizienten Nierenzellkarzinoms an. Dieses kann dann je nach weiterer Aufarbeitung in eine hereditäre oder sporadische Variante eingeteilt werden kann. Die vermehrte Diagnostik sporadischer Fälle hat einen Einfluss auf die Wahrnehmung der papillären NZK Typ 2. Man geht zunehmend davon aus, dass in der Gruppe der papillären Karzinome des Typs 2 unterschiedliche Subtypen existieren, was zu einer Revision der Nomenklatur dieser Typen führen könnte.

## Morphologisches Spektrum und Diagnostik

**Figure 1:**
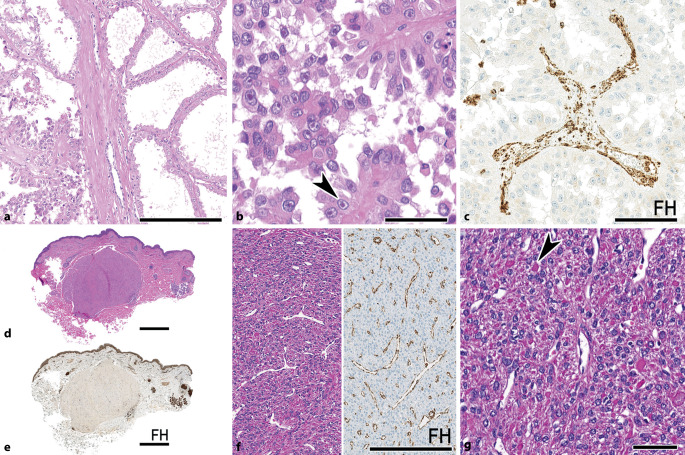

Das typische morphologische Muster des FH-defizienten NZK ähnelt häufig dem eines papillären Nierenzellkarzinoms (Typ 2). Es zeigen sich neben eosinophilem Zytoplasma klassischerweise sehr prominente eosinophile Nukleolen mit perinukleolärem Halo, die an virustypische Einschlusskörperchen erinnern (Abb. 1a, b). Hierbei sei allerdings zu erwähnen, dass diese Eigenschaft allenfalls sensitiv, nicht jedoch allzu spezifisch ist [8, 9]. Ein weiterer Punkt ist das Auftreten gemischter morphologischer Muster, die neben papillärer Differenzierung vor allem ein tubulozystisches (Abb. 1a, b), zystisches, kribriformes und/oder solides Wachstumsmuster zeigen können [2, 10]. Somit ist nicht verwunderlich, dass einige dieser Tumoren auch als unklassifizierte NZK, tubulozystische NZK oder auch Sammelrohrkarzinome [11] eingeordnet wurden. Insbesondere sei also hier auf die ungewöhnliche und auffällige Mischung verschiedener Differenzierung in dieser Entität hingewiesen [2]. Interessanterweise wurden unlängst auch einzelne FH-defiziente Fälle von low-grade onkozytär differenzierten Nierentumoren beschrieben, die morphologisch eher dem Spektrum SDHB-defizienter NZK zuzuordnen wären [12]. Ebenfalls haben wir kürzlich von ungewöhnlichen kolloidartigen zytoplasmatischen Inklusionen berichtet, die als möglicher Triggerpunkt für eine weiterführende Testung dienen können [13]. Hier zeigt sich die breite morphologische Varianz, die durch vermehrtes und besser zugängliches Testen mittels Immunhistochemie sicher noch nicht abschließend beschrieben ist. Die FH-Immunhistochemie bietet eine sehr spezifische Möglichkeit den Verlust der Expression darzustellen (Abb. 1c). Allerdings muss beachtet werden, dass vereinzelte Fälle gelegentlich keinen vollständigen Verlust von FH aufweisen und somit insbesondere schwierig zu diagnostizieren sind [8, 13]. Aufgrund der metabolischen Entgleisungen der Zelle kommt es zu einer vermehrten Succinierung von Proteinen, welche mit 2‑Succinocystein(2-SC)-Antikörper nachgewiesen werden kann [14]. Hierbei sei umgekehrt anzumerken, dass diese über eine sehr hohe Sensitivität, hingegen eine niedrigere Spezifität für die Diagnose eines FH-defizienten NZK verfügen [8]. Eine Kombination kann somit zur Erhöhung der Sensitivität in Betracht gezogen werden. In schwierigen Fällen ermöglicht jedoch nur eine molekularpathologische Untersuchung eine sichere Diagnose.

## Molekularpathologische Alterationen und ihre Folgen

Das *FH*-Gen codiert für das gleichnamige Protein, welches einigen noch aus dem Studium des Citratzyklus (bzw. Krebszyklus) bekannt sein dürfte. Dort katalysiert die Fumarat-Hydratase als Enzym die Reaktion, welche Fumarat in Malat umwandelt, und somit einen wichtigen Schritt in der mitochondrialen Energiegewinnung darstellt [15]. Sowohl hereditäre als auch sporadische Tumoren zeigen typischerweise einen vollständigen Verlust der FH-Expression durch eine biallelische Inaktivierung [7]. Eine Unterscheidung der Pathogenese (sporadisch vs. Keimbahn) ist mit diesem Surrogat somit nicht möglich. In der heterozygoten (hereditären) Konstellation wären dabei entweder ein Verlust („loss of heterozygosity“) des noch intakten Wildtypallels bzw. eine zweite (zusätzliche sporadische) pathogene Mutation auf dem (ursprünglichen) Wildtypallel denkbar. Kürzlich konnten wir zeigen, dass in verschiedenen Tumorentitäten innerhalb des gleichen Betroffenen mit dem HLRCC-Tumorsyndrom verschiedene dieser Mechanismen zu finden sein können [13]. Durch die Insuffizienz der enzymatischen FH-Reaktion kommt es zu einem konsekutiven Anstieg des sog. Onkometaboliten Fumarats, das nicht mehr weiter im klassischen Weg abgebaut werden kann. Dies führt zu vielfältigen und komplexen Veränderungen des Zellhaushaltes. Unter anderem sind eine Stabilisierung der „hypoxia inducible factors“ (HIF) sowie in der Folge ein Wechsel zur aeroben Glykolyse (sog. Warburg-Effekt), eine vermehrte Angiogenese via dem Vascular-endothelial-growth-factor(VEGF)-Signalweg, eine Alteration der mitochondrialen DNA sowie Inhibitionen der DNA-Reparatur beschrieben [15–17]. Hinsichtlich möglicher Therapien sind wenige, jedoch vielversprechende Daten mit der Kombination Erlotinib/Bevacizumab in der Literatur zu finden [13, 18, 19]. Die Seltenheit dieser Entität erschwert dabei prospektive Studien, zeigt hingegen jedoch auch die große Bedeutung einer korrekten Diagnose dieser molekular und metabolisch klar distinkten Tumorentität. Es werden dringend weitere Daten zu potenziellen Therapieoptionen dieser typischerweise hochaggressiven Tumorentität im jüngeren Lebensalter auf Basis des molekularen Wissens benötigt.

## Syndromale Assoziationen und ihre Identifikation

Genaue Daten zur Prävalenz des HLRCC-Syndroms sind nicht bekannt. Schätzungen deuten auf ca. 1 in 200.000 Individuen hin, bisher sind weltweit etwa 300 Familien beschrieben. Typischerweise präsentieren sich die Patienten bzw. Patientinnen in der 3. Lebensdekade mit kutanen (Abb. 1d, e) sowie uterinen (Abb. 1f, g) Leiomyomen [20]. Eine Screeningstudie auf FH-Defizienz zeigte dabei in uterinen Leiomyomen eine breite Schwankung von Prävalenzen, je nach morphologischem Spektrum. Konventionelle, unselektionierte Leiomyome zeigten dabei die niedrigste Rate (1,6 %), während morphologisch auffällige Varianten, wie z. B. atypische Leiomyome, eine deutlich höhere Prävalenz zeigten (37,3 %) [21]. Histomorphologische Studien identifizierten dabei rekurrente morphologische Muster, die auf FH-defiziente Leiomyome hinweisen können. Es finden sich gehäuft prominente Nukleoli mit perinukleolären Halos, hirschgeweihartig verzweigende Gefäße (Abb. 1f), eosinophile intrazytoplasmatische Globuli (Abb. 1g), Areale, die an ein alveoläres Ödem der Lunge erinnern, sowie ovoide Kerne mit schwannomartiger Palisadierung [21, 22]. Diese Veränderungen sind unter dem Begriff der sog. FH-defizienten Morphologie subsumiert. Ein immunhistochemischer FH-Expressionsverlust kann einen aufkommenden Verdacht untermauern, wobei wie auch in FH-defizienten NZK die Expression in einzelnen Fällen erhalten sein kann. Dies lässt sich zum Beispiel durch pathogene Mutationen erklären, die zu einem Funktionsverlust führen, allerdings das Epitop des verwendeten Antikörpers nicht betreffen [20]. Weiterhin eröffnet sich in der Folge die Schwierigkeit des korrekten klinischen Managements. Da aktuell nur wenige Fälle auf Keimbahnmutationen untersucht wurden, ist die Rate an sporadischen biallelischen Inaktivierungen des *FH*-Gens in FH-defizienten uterinen Leiomyomen mit großen Schwankungen beschrieben (von ca. 40 % bis 97 %), mit bis zu 50 % Keimbahnmutationen in Patientinnen unter 30 Jahren [22–24]. Es muss daher abgewogen werden, welche weiteren klinischen Maßnahmen zur Suche nach einer möglichen hereditären Assoziation ergriffen werden sollen. Dabei sollte auch in Betracht gezogen werden, dass die Leiomyome im Durchschnitt eine Dekade früher auftreten als die FH-defizienten NZK, die wiederum bei Diagnosestellung häufig bereits metastasiert sind und eine entsprechend ungünstige Prognose aufweisen [25]. Das Lebenszeitrisiko für Patienten mit nachgewiesenem HLRCC-Syndrom wird insgesamt auf etwa 15–20 % geschätzt [25, 26]. Bei nachgewiesener hereditärer Assoziation werden in der Literatur Screeningkonzepte zur Früherkennung von NZK diskutiert [26].

## Fazit für die Praxis

- Das Fumarat-Hydratase(FH)-defiziente Karzinom ist eine molekular/metabolisch distinkte Tumorentität, welche durch eine biallelische Inaktivierung des *FH*-Gens und konsekutivem Expressions- bzw. Funktionsverlust des Citratzyklusenzyms FH gekennzeichnet ist.
- Viruseinschlusskörperchenartige Nukleolen sind typisch, außerdem findet sich häufig eine gemischte Differenzierung mit papillären, tubulozystischen, kribriformen und/oder soliden Wachstumsmustern.
- Ein großer Anteil ist mit dem Hereditäre-Leiomyomatose-und-Nierenzellkarzinom(HLRCC)-Syndrom assoziiert, wobei auch ein zunehmender Anteil sporadischer Fälle beschrieben wird.
- Eine akkurate Diagnose kann durch immunhistochemischen Expressionsverlust von FH oder bei Verdacht und „erhaltener“ FH-Expression ggf. durch molekularpathologische Untersuchungen des *FH*-Gens im Tumorgewebe gestellt werden.
- Die korrekte Diagnose ist für ein optimales Management der Erkrankung von großer Bedeutung.
